# Altered resting-state functional connectivity of the salience network in patients with schizophrenia, with or without cannabis use disorder.

**DOI:** 10.1192/j.eurpsy.2025.525

**Published:** 2025-08-26

**Authors:** M. Matrone, G. Moltoni, A. Romano, G. Kotzalidis, A. Romano, P. Motta, F. Nicoletti, S. De Persis, A. Bozzao

**Affiliations:** 1 Neurosciences, Mental Health, and Sensory Organs Department (NESMOS), Sapienza University of Rome, Faculty of Medicine and Psychology, Rome; 2 Department of Mental Health Protection and Promotion, Unit of Addiction Pathology, Rieti; 3 Department of Physiology and Pharmacology, Sapienza University of Rome, Rome, Italy

## Abstract

**Introduction:**

Schizophrenia (SCZ) is a mental disorder with as yet undefined aetiology and pathogenesis. It is currently considered as a neurodevelopmental disorder and one of the main causes of disability globally, with a prevalence of approximately 1%. About one in four individuals with SCZ is diagnosed with comorbid Cannabis Use Disorder (CUD). Aberrations in brain connectivity have been identified as contributing to the pathophysiology of SCZ.

**Objectives:**

To our knowledge, no resting-state functional magnetic resonance imaging (rs-fMRI) study has compared continued cannabis use in SCZ patients with CUD (SCZ-CUD^+^) *vs.* patients with SCZ without cannabis use (SCZ-CUD^-^). We hypothesised that continued cannabis use, could result in greater impairment of clinical and cognitive symptoms and in altered connectivity in the Salience Network (SaN), in striate/extended amygdala-cortical areas, and in those involved in planning and emotional control. Studying connectivity in patients with SCZ and CUD could elucidate the mechanisms underlying the development of psychotic symptoms and vulnerability to substance use relapse.

**Methods:**

We included 14 SCZ-CUD^+^ and 20 SCZ-CUD^-^ patients. All were assessed cross-sectionally through the Neurological Evaluation Scale, the Brief Assessment of Cognition in Schizophrenia, the Positive And Negative Syndrome Scale and the Clinical Global Impressions Scale-Severity, and underwent brain rs-fMRI with a 1.5 T scanner to explore functional connectivity (FC). *Imaging protocol:* All patients underwent a brain 1.5 T MRI scan (GE Signa Voyager) with 32-channel phased-array head coil. Beside the conventional morphological sequences, the functional MRI protocol included: 1) rs-fMRI obtained with a gradient-echo echo-planar imaging (EPI) sequence. Each scan session lasted for 6 min. 2) Structural imaging involved a sagittal three-dimensional sequence employing a magnetisation prepared rapid gradient echo (MPRAGE) over the whole brain.

**Results:**

CUD in SCZ patients was associated with higher impulsiveness and excitement, and lower negative symptoms and neurological soft signs (Fig.1). SCZ-CUD^+^ and SCZ-CUD^-^ groups differed little on cognitive performance, except for Symbol Coding, where SCZ-CUD^-^ outperformed SCZ-CUD^+^. Rs-fMRI, compared to SCZ-CUD^-^, showed reduced FC in patients with SCZ-CUD^+^ between regions of the salience network, including anterior cingulate and prefrontal cortices, and between right insula and dorsolateral prefrontal cortex and precuneus (Fig.2, Fig.3).

**Image 1:**

**Figure:**
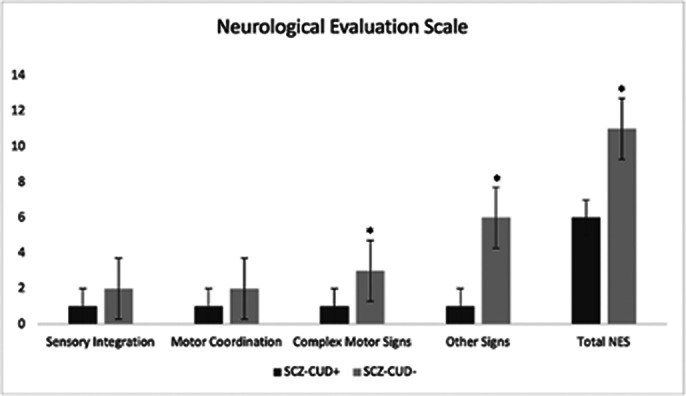

**Image 2:**

**Figure:**
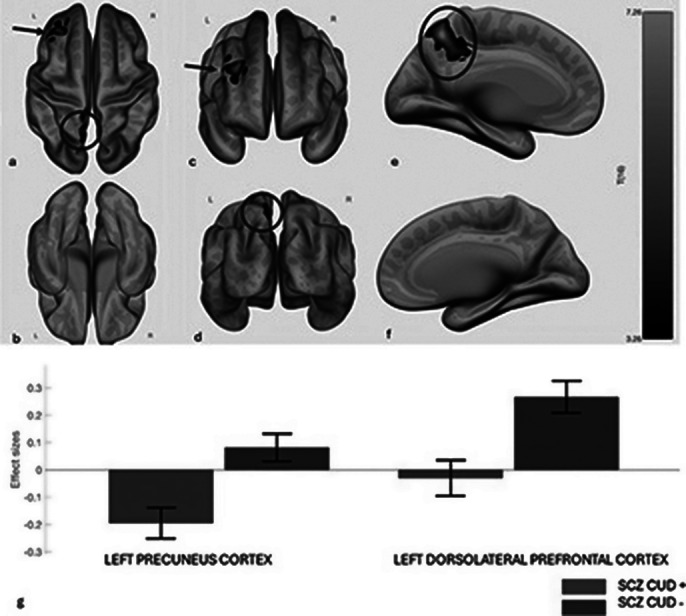

**Image 3:**

**Figure:**
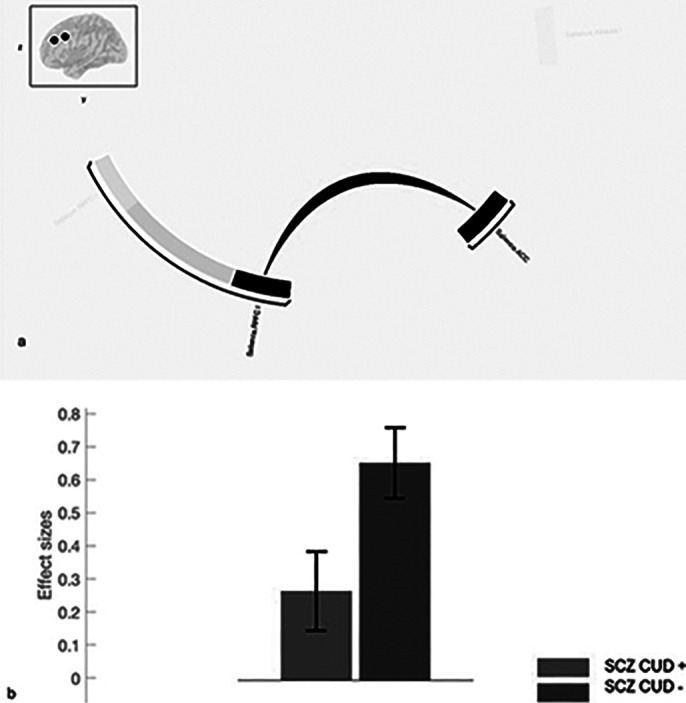

**Conclusions:**

Using rs-fMRI, we revealed differences in FC within the SaN between SCZ-CUD^-^ and SCZ-CUD^+^. Continuous cannabis use reduces FC in cortical circuits, impacting connections already impaired in SCZ. In SCZ-CUD^+^ patients, altered self-relevance attribution and insular dysfunction may increase drug-related impulses and impair cognitive control.

**Disclosure of Interest:**

None Declared

